# Off-Label Use of an External Hand Fixator for Craniomaxillofacial Fractures—An Anatomical Feasibility Study

**DOI:** 10.3390/bioengineering11030279

**Published:** 2024-03-15

**Authors:** Florian Wichlas, Marco Necchi, Teresa Gruber, Valeska Hofmann, Susanne Deininger, Sebastian Hubertus Markus Deininger, Amelie Deluca, Eva Steidle-Kloc, Jan Pruszak, Jörn Wittig, Christian Deininger

**Affiliations:** 1 Department of Orthopedics and Traumatology, University Hospital Salzburg, Paracelsus Medical University, Müllner Hauptstrasse 48, 5020 Salzburg, Austria; fwichlas@nolimitsurgery.com (F.W.); teresa.gruber@stud.pmu.ac.at (T.G.); christian.deining@hotmail.com (C.D.); 2 No Limit Surgery, Ernest-Thun-Strasse 6, 5020 Salzburg, Austria; marco.necchi@hotmail.it (M.N.); vhofmann@nolimitsurgery.com (V.H.); s.deininger@salk.at (S.D.); s.deininger@hotmail.com (S.H.M.D.); 3 Department of Surgery and Orthopaedics, Hospital Sterzing, Margarethenstraße 24, 39049 Sterzing, Italy; 4 BG Trauma Centre, Department of Trauma and Reconstructive Surgery, University of Tübingen, 72076 Tübingen, Germany; 5 Department of Urology and Andrology, University Hospital Salzburg, Paracelsus Medical University, Müllner Hauptstrasse 48, 5020 Salzburg, Austria; 6 Institute of Tendon and Bone Regeneration, Paracelsus Medical University, 5020 Salzburg, Austria; 7 Institute of Anatomy and Cell Biology|Salzburg, Paracelsus Medical University, 5020 Salzburg, Austria; eva.steidle@pmu.ac.at (E.S.-K.); jan.pruszak@pmu.ac.at (J.P.); 8 Center of Anatomy and Cell Biology, Salzburg and Nuremberg, Paracelsus Medical University, 5020 Salzburg, Austria; 9 Department of Oral and Maxillofacial Surgery, University Hospital Salzburg, Paracelsus Medical University, Müllner Hauptstrasse 48, 5020 Salzburg, Austria; j.wittig@salk.at

**Keywords:** biomechanical evaluation, craniomaxillofacial fracture, CT scan, external fixator, feasibility study on the cadaver, low-income countries

## Abstract

Background: The lack of resources limits the treatment of craniomaxillofacial fractures (CMF) in low-income countries (LIC). Therefore, Barton bandages and/or interdental wiring are considered in these regions. Fracture reduction is maintained by permanent occlusion for 6 weeks, which often leads to limited compliance and dissatisfying results. The aim of this cadaver-based study is to evaluate the feasibility of the use of an external face fixator (EFF) for the treatment of CMF, its biomechanical values and to define the optimal pin insertion points and angles. Materials and Methods: An AO hand fixator was used. CMF of types Le Fort 1–3 with split fractures of the hard palate were treated with EFF on 13 anatomical specimens. Fractures were created using a chisel, and pins were placed in specific anatomical regions. The maximal pull-out force [N] of pins was analysed by a tensile force gauge, and Fmax of the mandibular pins was evaluated. Computer tomography scans were performed on the healthy, fractured and EFF-treated skulls. Results: The pull-out forces for the single pins were mandibular pins (n = 15, median 488.0 N), supraorbital pins (n = 15, median 455.0 N), zygomatic pins (n = 14, median 269.1 N), medial hard palate pins (n = 12, median 208.4 N) and lateral hard palate pins (n = 8, median 49.6 N). Conclusions: The results indicate that the operation technique is feasible, and the stability of the EFF is sufficient for maintaining the reduction. The required pins can safely be inserted into the described areas with good reduction results. Using EFF offers a feasible alternative to the non-surgical treatment of CMF in LIC.

## 1. Introduction

Craniomaxillofacial fractures (CMF) are common in low-income countries (LIC) due to the lack of passive safety measures such as motorcycle helmets or airbags [1,2,3,4,5]. The gold standard of treatment for these fractures in a high-income country (HIC) is open reduction and rigid internal fixation by miniplate osteosynthesis of the facial bones [6,7]. This requires a pre-operative computed tomography (CT) scan or cone beam CT to plan the surgical technique, the necessary osteosynthesis equipment and a capable surgeon. The majority of midfacial fractures will be missed with conventional X-rays [8,9,10]. Some clinics even use an intraoperative 3-D CT scan to verify the reduction [11,12,13]. While surgical care for CMF in HIC has changed and improved significantly over the past 100 years, there have been few improvements in LIC [14]. In LIC, 3D scans or material for osteosynthesis are often not available. CMF are therefore frequently treated non-operatively with a Barton bandage, suspension wiring to the upper face or skull or interdental wiring [15]. These treatment approaches usually lead to acceptable results and good occlusion, if the patient is compliant [16]. The Barton bandage or maxillomandibular fixation is applied to maintain the reduction of the fracture by rigid occlusion for 6 weeks. Ingestion is only possible with fluids, and speaking as well as oral hygiene are significantly impeded. Compliance is correspondingly low [17,18].

A possible solution to this treatment deficit in LIC is the external face fixator (EFF) as an alternative tool for the definitive treatment of CMFs. This can be applied without a pre-operative CT scan, is usually available in LIC as a widespread hand fixator system is used and allows dynamic occlusion over the 6-week treatment period. In addition, it enables almost normal oral hygiene and speech. Soft food can be eaten. Meanwhile, more than 30 patients with CMF were treated with the EFF in a hospital in Sierra Leone, Africa [19].

The aim of this study on the anatomical specimen was to provide detailed surgical instructions for applying the EFF and a biomechanical evaluation of pin stability. For this purpose, the reduction of the CMF with the EFF was analysed evaluating pre- and post-operative CT scans. Thus, the optimal angle of the pins for the best intraosseous drilling depth could be defined. After application of the EFF to the anatomical specimen, the areas and the endangered structures were dissected.

To provide a detailed overview of the anatomical conditions in the area of the insertion sites, digital measurements of different angles and corresponding possible intraosseous drilling depths were carried out bilaterally on 100 clinical CT scans of the skull of healthy subjects.

The goal is to bring this alternative treatment option for CMF in LIC from bench to bedside to improve surgical care in these regions of the world.

## 2. Materials and Methods

All experiments were performed in accordance with relevant guidelines and regulations of the Ethics Commission (EK No: 1198/2021). All body donors presented in the manuscript provided informed consent for the scientific use of their bodies in writing and before their death occurred. The work has been reported in line with the Standards for Quality Improvement Reporting Excellence (SQUIRE) criteria [20].

### 2.1. Tests on Anatomical Specimens

A commercially available AO hand fixator (DePuy Synthes, Raynham, MA, USA) was used for the EFF [21]. Pins were selected in diameters of 3.5 and 4.0 mm. The clamping jaws and carbon rods were available in standard dimensions.

A CT scan was performed of 3 cadaver skulls in the following settings: non-fractured, after setting the fractures and after applying the EFF and fracture reduction (Computed tomography: Somatom Emotion 6, Siemens Healthineers, Erlangen, Germany).

The determination of the maximum pull-out force was carried out by a calibrated tensile force gauge (Sauter FK 500, Fmax = 500 N, Erlangen, Germany). The anatomical specimen was fixed tightly to the table with tension straps. The device was attached to the respective pin with an angle-stable hook system and pulled in the orthograde direction. The pull-out forces were measured for the palatal pins, the pins in the zygomatic bone and the supraorbital pins. As a reference, a mandibular fixator was also attached and the pull out forces for each pin separately measured, since this technique is widely established [22].

CMFs were set with a chisel on 13 anatomical specimens in the sense of a combination of a Le Fort 1, 2 and 3 fracture with a palatal split and a naso-orbitoethmoid fracture as this combination of fractures is common in LIC [23,24,25,26]. Primarily, the hard palate was split longitudinally, and a Le Fort 1 fracture was performed. Then the anterior sinus wall and nasofrontal suture were fractured on both sides like a Le Fort 2 fracture. Finally, a fracture of the lateral orbital rim and the zygomatic arch were placed on both sides in the sense of a Le Fort 3 fracture.

After setting the fractures, the EFF was applied. The reduction was performed bottom-up. Primarily, the palatal pins were inserted, and a crossbar was attached to close the palatal split. If a rotational instability was still present, or the placement of lateral palatal pins was unsatisfactory, a 2.0 mm Kirschner wire was inserted behind the cuspid of the canines or 1st premolar teeth, bridging the split to improve the rotational instability of the construct. Subsequently, two pins were inserted into the zygomatic bones and connected to the first rod. Finally, the supraorbital pins were placed on both sides. The final step was the biomechanical evaluation of the pull-out forces for the individual pins.

### 2.2. Application of the EFF Step by Step

The self-drilling pins were inserted after performing a stitch incision with a scalpel, blade size 11.

#### 2.2.1. Reduction and Fixation of the Palatal Split

**Figure 1:**
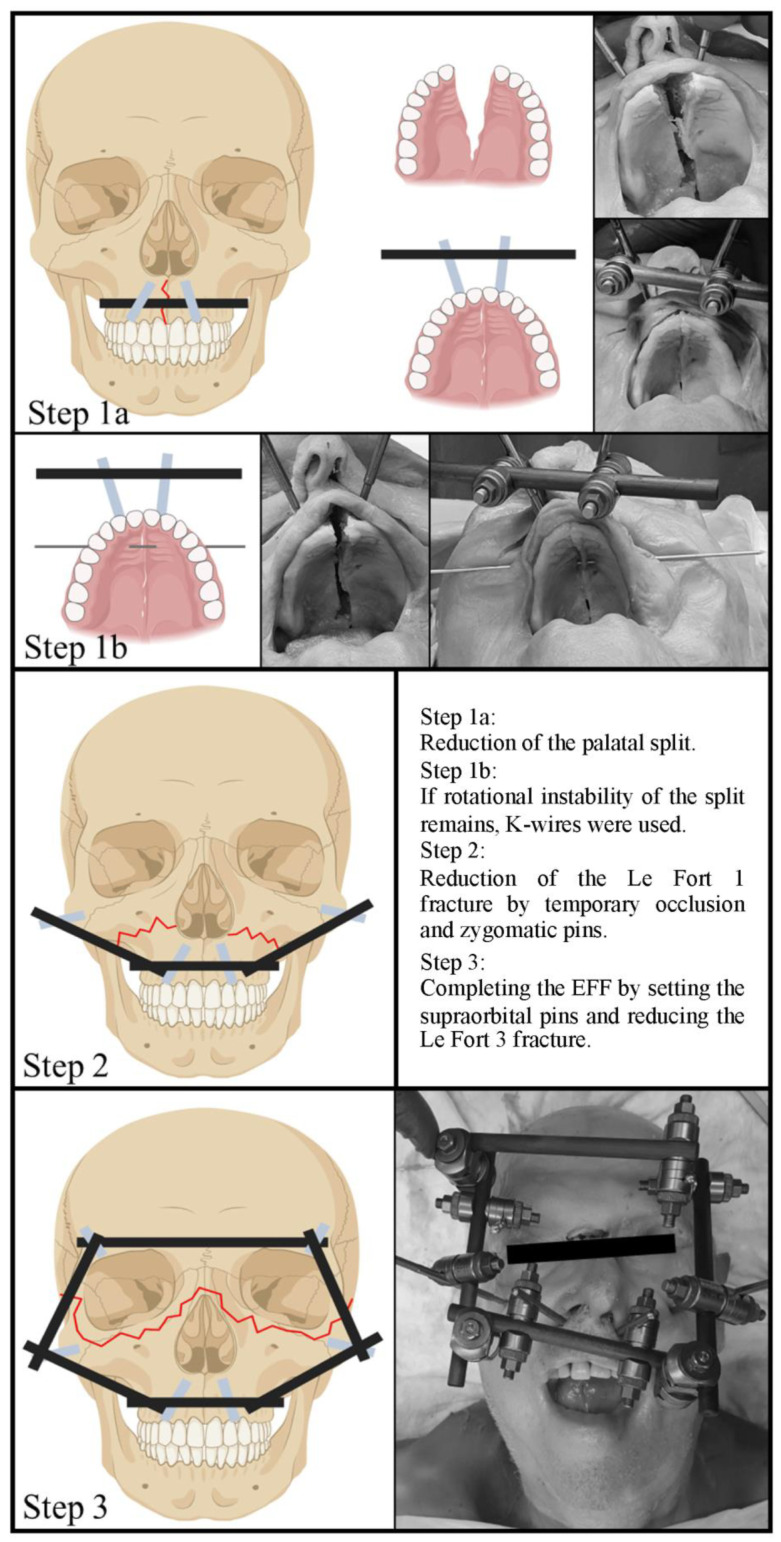
Fracture reduction and applying of the External Face Fixator step by step.

Primarily, the reduction of the palatal split was performed. For this purpose, two medial palatal pins were inserted into the hard palate, directly below the nostrils on both sides. They were drilled until the tip could be palpated with the finger under the oral mucosa. The pins were connected to a carbon rod and, after the palatal split had been reduced with the finger under sight and control, were turned tight (Figure 1). If the pins in the lateral hard palate showed decreased clinical stability, a K-wire osteosynthesis was inserted as an alternative in combination with the pins in the medial hard palate to increase rotational stability (Figure 1).

#### 2.2.2. Reduction and Fixation of the Le Fort 1 and 2 Fracture Components

If the zygomatic bone was not affected by a comminuted fracture, one pin was inserted into it on both sides and the pins were connected to the transverse carbon rod from step one. The reduction was achieved by temporary occlusion. In this position, the clamping jaws were turned tight. The reduction was checked clinically.

#### 2.2.3. Reduction and Fixation of the Le Fort 3 Fracture Component

As a last step, the EFF was completed by inserting the supraorbital pins in the lateral third of the upper orbital margin. After inserting the pins, they were connected to one another with a rod and to the carbon rod of the zygomatic pins. After reduction of the Le Fort 3 fracture component, the clamping jaws were tightened (Figure 1). If the zygomatic pins could not be inserted because of a comminuted fracture of the bone, the reduction was achieved by temporary occlusion at this step.

### 2.3. Analysis of the Optimal Insertion Angle for the Longest Intraosseous Drilling Possibility

To give a detailed impression of the bony condition for the supraorbital pin insertion of the EFF with optimal insertion angles and the corresponding possible intraosseous drilling depth, the supraorbital areas of 100 CTs of the skull of healthy subjects were digitally determined.

**Figure 2:**
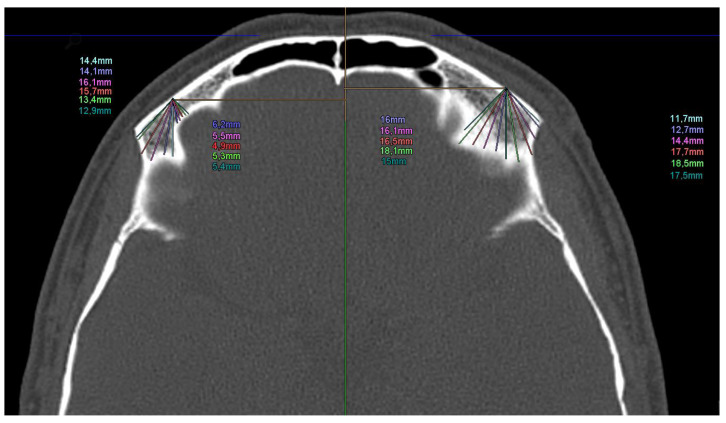
Axial section of a clinical CT scan at the level of the supraorbital insertion area with the measured angles and corresponding potential monocortical drilling depth.

The measured values of the angles and possible intraosseous drilling depth are shown in Figure 2.

### 2.4. Digital Measurement of CT Scans for Analysis of Optimal Pin Insertion Angles

To define the optimal insertion angles of the supraorbital pins, digital analysis of a clinical dataset of 100 healthy CT scans of the skull was performed (age: 33.4 ± 9.7 years). A central line was drawn in an axial slice, and the insertion site for supraorbital pins was determined in the lateral third of the os frontalis pars orbitalis. Subsequently, the possible intraosseous drilling depth was measured at different angles (−45°, −40°, −30°, −20°, −10°, 0°, 10°, 20°, 30°, 40°, 45°). The percentile analysis is used to determine the optimal surgical angle. In a second step, we examined the distribution of the optimal and worst surgical angles using a histogram. The goal is to determine the optimal and unfavourable window for the insertion angle seen over all patients.

A skull was anatomically dissected postoperatively and after removal of the EFF by an anatomist to identify structures at risk and document any damage.

### 2.5. Statistical Methodology

To show the differences in the pull-out force among the different pin insertion areas, an ANOVA analysis with Kruskal–Wallis and Dunn’s multiple comparison tests was carried out. To determine the optimal insertion angle, we use simple statistical methods, such as percentile analysis and histograms.

## 3. Results

### 3.1. The Biomechanical Evaluation of the Pull-Out Force of the Pins

**Figure 3:**
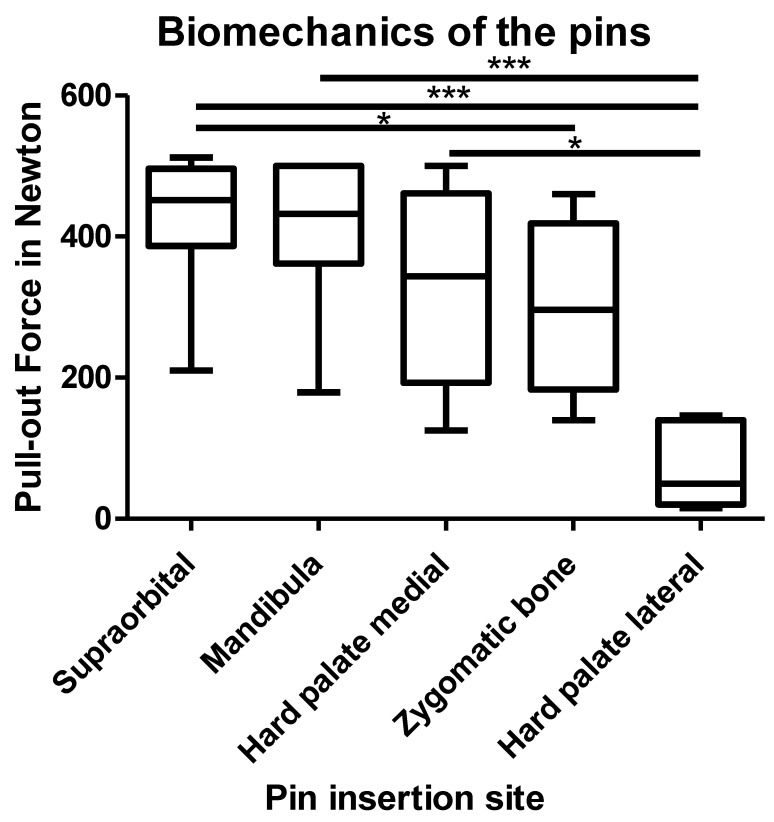
Evaluation of the pull-out forces of the EFF pins using an ANOVA analysis with Kruskal–Wallis and Dunn’s multiple comparison tests. Significance level: (*** *p* < 0.001, * *p* < 0.05). All other pull-out forces were not significantly different.

The pins were pulled individually and in orthograde direction to a force up to 500 N. The pull-out forces of the pins in the mandibula were set as a reference value. The measured values for the individual pins are shown in Figure 3. After completing the entire EFF, the design could not be pulled out by hand.

There were significant differences in the stability of the individual areas. The post-hoc Dunn’s multiple comparison test showed the following values for the pull-out tests for the individual pin areas: The supraorbital pins showed the greatest stability, followed by the mandibular pins and the pins in the medial hard palate. The pins in the zygomatic bone showed a significantly lower pull-out force compared to the supraorbital pins and the pins in the medial hard palate (*p* < 0.05). The lowest pull-out force could be measured with the pins in the lateral hard palate. Here, a highly significant reduction in the holding force compared to the supraorbital pins and the pins in the mandibula was shown (*p* < 0.001). The measured maximum pull-out forces can be seen in Table 1, the ANOVA analysis with Kruskal–Wallis and Dunn’s multiple comparison tests can be found in Table 2.

### 3.2. Results of the Digital Evaluation of the Possible Pin Insertion Areas of 100 CTs of the Skull

The supraorbital and zygomatic pins were inserted in the sagittal plane at 0° deviation. The pins in the medial hard palate were oriented perpendicular to the curvature of the hard palate until the tips of the pin could be palpated with the finger under the oral mucosa.

The optimal drilling angle to achieve the longest and safest possible intraosseal canal was determined in the axial plane using 100 cranial clinical CT scans.

As shown in Figure 2, the supraorbital pins provide the highest stability of the EFF in the pull-out test. However, there is a risk of drilling too deep or in a non-optimal angle in this region and thus drilling into the anterior cranial fossa. To prevent this and a potential injury to the brain or subsequent infection of the central nervous system, the optimal drilling angles and their inter-individual deviation were analysed based on 100 skull CT scans.

**Figure 4:**
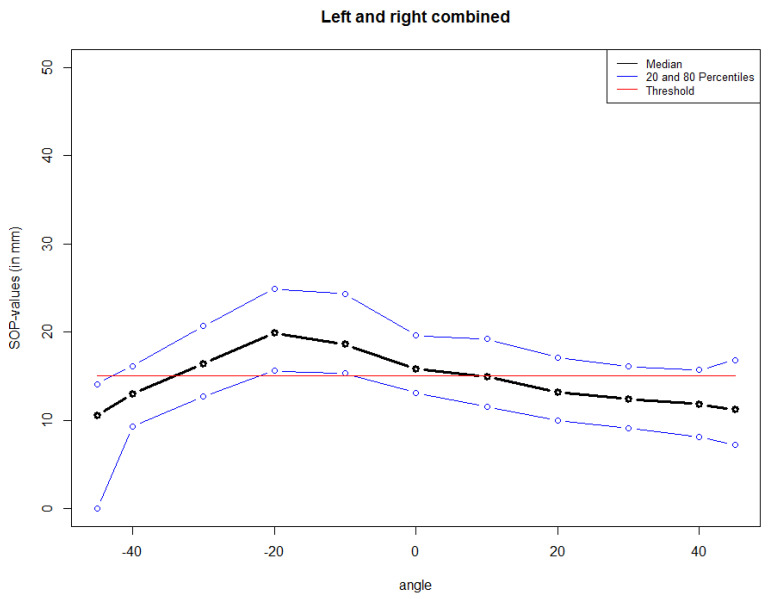
Median and 20/80 percentiles of the supraorbital pins by intersection angle with 15 mm possible intraosseous drilling depth.

Referring to Figure 4, the optimal intersection angle for the supraorbital pins is −10°, followed by −20°. In both cases, at least 80 percent of patients showed scan values above the previously defined threshold value (15 mm).

**Figure 5:**
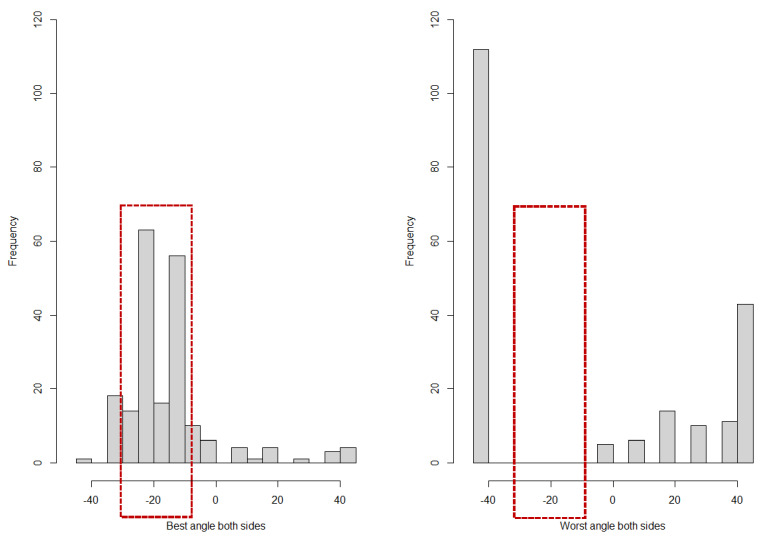
Histograms of the best and worst angles found. A relative majority of patients show best angle values in the range of −15° to −10°, followed by −25° to −20° (red frame). In general, the majority of best angle values are in the range of −35° to −10°.

Figure 5 illustrates histograms of the best and worst angle found for each patient. It confirms that a relative majority of patients showed best angle values in the range of −15° to −10°, followed by −25° to −20°. In general, the majority of best angle values were in the range of −35° to −10°. For the worst angle, on the other hand, all patients show values below −40° or above −5°. In other words, there is no patient with a worst angle in the range that is considered optimal, referring to the findings shown in Figure 5.

The results of a linear regression showed no significant influence of sex, age or both indicators combined on the best or worst intersection angle.

### 3.3. Clinical Evaluation of the Surgical Method

The EFFs were applied by three trauma surgeons. Two of them had already applied EFFs in Sierra Leone in the past, and one had previously been instructed on anatomical specimens. Subjectively, the surgical method was feasible. The reduction of the palatal split and the Le Fort components was clinically achievable. The insertion of the pins in the above-mentioned areas was similar to that of a fixator of the extremities. In the supraorbital region, it was always drilled monocortically, and in the other regions, bicortically. The pin in the lateral hard palate showed clinically insufficient stability. When inserting the pin into the medial hard palate, the drilling direction was determined by palpating the tip of the pin with the finger under the oral mucosa at the hard palate.

### 3.4. Postoperative Results in the CT Scans

**Figure 6:**
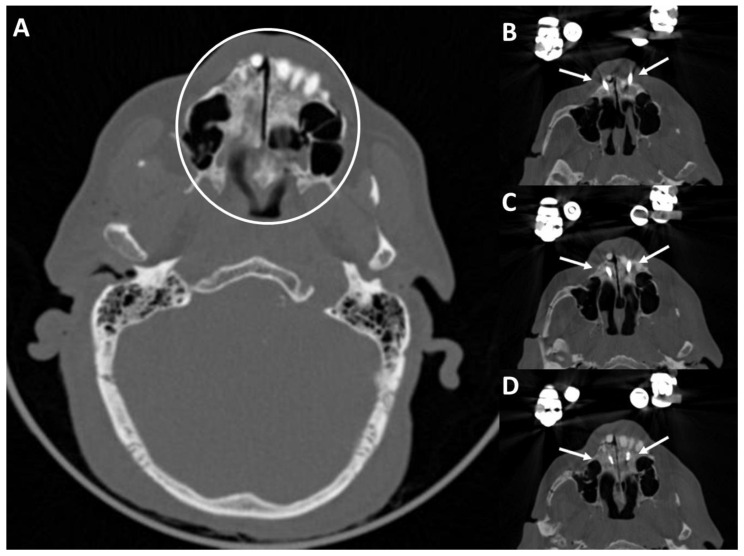
CT scans of the fractured skull (**A**) and palatal split (white circle). (**B**–**D**) Reduced palatal split with two medial palatal pins (white arrows).

Figure 6 shows the fracture situation with palatal split in the axial cross-sectional plane and the reduction result achieved with the two pins in the medial hard palate. The drilling depths of the pins can be seen on the right side. The fracture is reduced in the post-operative imaging.

**Figure 7:**
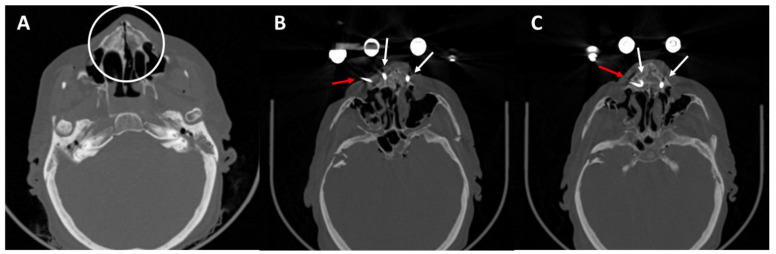
CT scans of the fractured skull (**A**) and palatal split (white circle). (**B**,**C**) Reduced palatal split. K-wire (red arrow) and two medial palatal pins (white arrows).

The biomechanical examination showed significantly reduced stability of the pins in the lateral hard palate. In order to address a slight rotational instability of the hard palate in the intraoral direction, despite inserted and connected medial pins, an additional K-wire osteosynthesis was performed. This led to a good reduction result in the postoperative CT scan (Figure 7).

**Figure 8:**
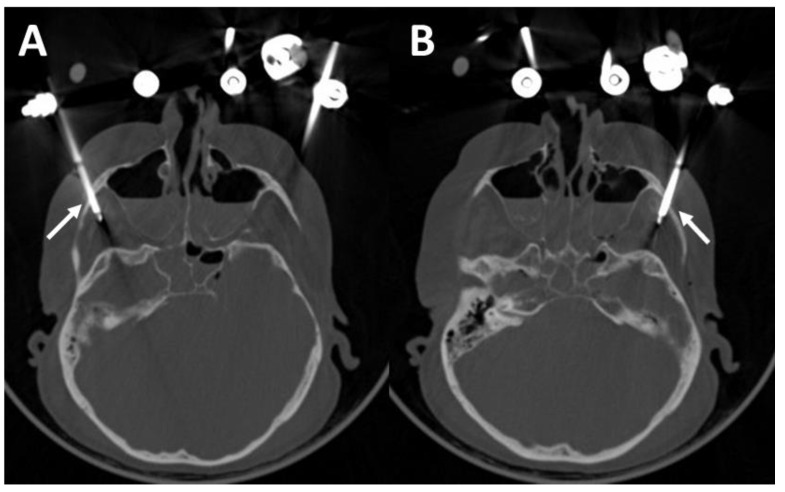
CT scans of the fractured skull with right (**A**) and left and right (**B**) zygomatic pins (white arrows).

The zygomatic pins are shown in axial cross section in Figure 8. Although the pins protrude into the infratemporal fossa, they showed adequate stability in the biomechanical examination.

**Figure 9:**
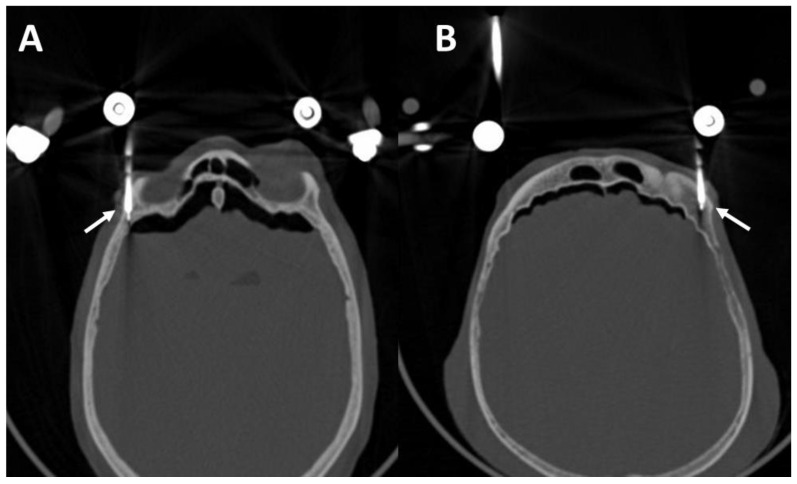
CT scans of the fractured skull with right (**A**) and left and right (**B**) supraorbital pins (white arrows).

A postoperative CT scan of the monocortically drilled supraorbital pins is shown in Figure 9. Both pins are inserted intraosseously without penetrating the anterior cranial fossa.

### 3.5. Anatomical Dissection of the Pin Insertion Areas and Description of Structures at Risk

**Figure 10:**
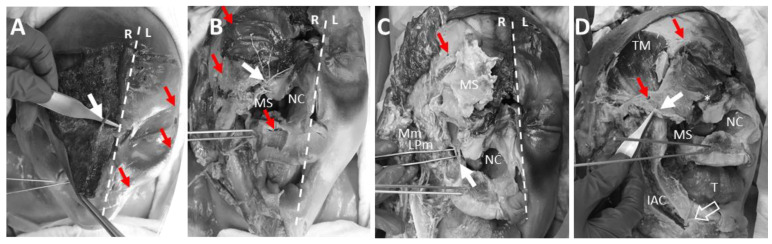
Photodocumentation of the dissection site with white dashed lines separating the face into a dissected halve (right, R) and a non-dissected halve (left, L). Red arrowheads document the insertion sites of the fixature pins. (**A**) Fronto-cranial view: arrow marks the supraorbital nerve; (**B**) Frontal view: arrow points to the infraorbital nerve leaving the infraorbital foramen (with infraorbital artery removed); NC = opening of the nasal cavity; MS = opening of the maxillary sinus; (**C**) Frontal view: arrow marks the superior alveolar nerve and artery; LPm = lateral pterygoid muscle; Mm = masseter muscle; MS = maxillary sinus (frontal bone of the maxillary sinus folded up); NC = opening of the nasal cavity; (**D**) Right lateral view: the transverse facial artery, asterisk marks the infraorbital foramen, open arrow marks the mental nerve (mental artery was dissected); IAC = the opened inferior alveolar canal with the inferior alveolar nerve and the inferior alveolar artery; NC = nasal cavity; MS = maxillary sinus; T = tongue; TM = temporalis muscle.

One side of the face was anatomically dissected. Within the superior orbital region, the supraorbital nerve (Figure 10A) was found not to be affected by the pin inserted lateral to the nerve. The supratrochlear nerve might be affected by the trauma but not the pin. This might also be the case for the infratrochlear nerve at the medial border of the orbit. The infraorbital nerve (Figure 10B) and infraorbital artery were also not affected by the more laterally inserted zygomatic pin or the caudally inserted palatine pin, but might be affected by the trauma since lesions in the infraorbital region were found to be very distinct in this specimen (Figure 10C).

The transverse facial artery was intact as well as the zygomatico-orbital artery. The temporal branches of the facial nerve were shown to not be affected by the inserted pin in this specimen. The zygomatic branches of the facial nerve could not be clearly identified during the dissection according to the trauma. It has to be noted that this structure is at risk to be damaged by insertion of the zygomatic pin.

Within the dissected specimen, the maxillary sinus was severely damaged in line with the created fractures (Figure 10D). The superior alveolar nerve and artery were not affected neither by the fracture itself nor the inserted pins in this specimen; however, smaller distal branches of these structures may be at risk. The palatal bone pins also did not affect any visible structures in the dissected specimen. When placing the K-wire, dental nerve roots can be affected. Singular dental nerve roots of the frontal to the canine teeth were not dissected and checked for damage. The inferior alveolar nerve and artery also remained unaffected as was the mental nerve.

## 4. Discussion

A glaring gap still exists in the care of patients with CMF in LIC compared to the treatment options in HIC [27]. To address this gap and to improve the medical care quality, special teaching programs have been established to train resident surgeons in the care of these severe injuries [28,29]. However, this is only one essential component for the treatment of these fractures. In addition, preoperative CT scans are needed to plan the procedure, as well as the necessary instrumentation. Both are still not available in most remote hospitals in LIC [16]. An alternative is to transfer the patient to a better-equipped hospital with the necessary resources. However, this delay of care often leads to an increase in complications and poorer outcomes [30]. Porter et al. showed in a retrospective data analysis published in 2013 that CMF in South Africa were treated an average of 20.4 days after the accident. It took an average of 10.3 days for the patient to be admitted to a central hospital further away and a similar amount of time for surgical treatment to be performed [31]. An additional challenge is that in some of the poorest countries in the world, there is no adequate care centre at all. Other research groups have already demonstrated the benefit of surgical techniques developed in LIC due to a lack of resources [32].

The standard treatment in HIC before introduction of miniplate osteosynthesis consisted of archbars, craniofacial suspension wiring and maxillomandibular fixation. Palatal fractures are usually addressed by palatal plates that are wired to the teeth. To achieve good results with these techniques, a trained surgeon who is used to work intraorally is needed. The patient has to be dentate or needs adequate dentures. Furthermore, the patient has to be nourished with an adequate liquid diet for 6 weeks. All these factors can prove difficult in LIC, and therefore many of these patients remain untreated. The aim of this study is to objectively analyse a possible alternative for the treatment of CMF in LIC on anatomical specimens.

For completeness, it should be pointed out that secondary trauma after EFF can lead to intracranial pin migration. This has to be explained to the patient.

### 4.1. The Surgical Technique

The application of the EFF was feasible by the trauma surgeons. Reduction was achieved by temporary occlusion and simultaneous tightening of the EFF. The insertion of the bicortical pins (medial hard palate) and the monocortical pins (supraorbital) was technically feasible. It is comparable to the insertion of pins at the extremities.

### 4.2. Post-Operative CT Scans

Good reduction results were seen in the postoperative CT scans. In particular, the palatal split was closed and the maxillary fracture extending intraorally restored. The position of the pins in the imaging was also as planned. In particular, the position of the supraorbital pins appeared adequate. There was no evidence of injury to the internal cranial cortex. The pins in the medial hard palate ended clinically under the mucosa and showed good stability in biomechanical testing.

### 4.3. Biomechanical Analysis

To verify the stability of the pins, pins for a mandibular fixator were used as a reference. This is already clinically established and leads to good clinical results and sufficient stability. In the biomechanical examination, the supraorbital pins even showed a better hold than those in the mandible. The pins in the medial hard palate as well as in the zygomatic bone were also stable. In the statistical evaluation, there was no significant difference among the pins. It can be assumed that the selected pin insertion sites lead to sufficient stability. Due to the significantly lower stability of the pins in the lateral hard palate, a K-wire was inserted as an alternative. This appears to be particularly necessary in the presence of a split of the hard palate. Clinically, when only the two pins in the medial hard palate were inserted, this still showed a slight residual rotational instability, which could be stabilized by the K-wire.

The intraosseous drilling depth of the supraorbital pins was measured. There was no statistically significant correlation between the intraosseous drilling depth and the maximum pull-out force. A possible explanation for this could be that the bone substance has more influence on the pull-out force than the different drilling depths. Since patients with CMF in LIC are mainly young, healthy individuals, the sufficient drilling depth was set at 15 mm.

### 4.4. Evaluation of the 100 CT Scans of Healthy Subjects

The aim of this evaluation was to determine the range of optimal angles for supraorbital pin insertion using a data set of young, healthy subjects (Figure 3). The pins should reach as wide a distance intraosseous as possible without crossing the contralateral cortex to intracranial. Based on the data from the experiment on the anatomical specimen, a drilling depth of 15 mm was chosen. It was found that the optimal intraosseous drilling depth was possible in the range of −35° to −10° in more than 80% of the subjects.

### 4.5. Anatomical Dissection of the Pin Insertion Sites

Postoperatively, anatomical preparation of a skull was performed. The aim was to identify structures at risk and to document any injuries. No injuries of vessels or nervous structures by the pins could be detected. Injury to tooth roots cannot be ruled out when the K-wire is used to stabilize the palatal split, but this risk was accepted to prevent split rotation and may be negligible in severe trauma as the patients often lose the front row of teeth.

## 5. Conclusions

EFF represents an alternative treatment option to the non-operative approach for CMF in LIC. The surgical method can be performed safely and does not require a pre-operative CT scan or expensive mini-plates. In addition, the required instruments are usually also available in LIC, and trauma surgeons are practiced in handling them. Therefore, it can improve the care of patients in LIC.

## Figures and Tables

**Table 1:** List of the measured values of the pull-out tests of the individual 5 pin areas with accompanying descriptive statistics in Newtons. Decreasing from left to right, the median value in bold.

Pin Insertion Areas:	Supraorbital	Mandibula	Hard Palate Medial	Zygomatic Bone	Hard Palate Lateral
Number of values	21	16	14	13	8
Force in Newton:					
Minimum	210.0	179.0	125.0	139.8	15.00
25% Percentile	386.8	362.0	192.5	183.3	20.05
Median	451.8	432.0	343.8	296.0	49.60
75% Percentile	496.0	500.0	461.3	418.5	139.8
Maximum	512.0	500.0	500.0	460.0	146.0
Mean	428.4	415.3	326.6	293.8	72.23
Std. Deviation	80.38	94.99	139.8	115.8	59.26
Std. Error	17.54	23.75	37.37	32.12	20.95
Lower 95% CI of mean	391.8	364.7	245.9	223.8	22.68
Upper 95% CI of mean	464.9	465.9	407.3	363.8	121.8

**Table 2:** Significance level: (*** *p* < 0.001, * *p* < 0.05). Total significance level: **** *p* < 0.0001. All other pull-out forces are not significantly different. ns = not significant.

Kruskal–Wallis Test			
***p* value**	**<0.0001**		
Exact or approximate *p* value?	Gaussian Approximation		
*p* value summary	****		
Do the medians vary signif. (*p* < 0.05)	Yes		
Number of groups	5		
Kruskal–Wallis statistic	31.01		
Dunn’s multiple comparison test	Difference in rank sum	Significant? *p* < 0.05?	Summary
Supraorbital vs. Mandibula	1.772	No	ns
Supraorbital vs. Hard palate medial	15.07	No	ns
Supraorbital vs. Zygomatic bone	21.31	Yes	*
Supraorbital vs. Hard palate lateral	42.80	Yes	***
Mandibula vs. Hard palate medial	13.30	No	ns
Mandibula vs. Zygomatic bone	19.54	No	ns
Mandibula vs. Hard palate lateral	41.03	Yes	***
Hard palate medial vs. Zygomatic bone	6.242	No	ns
Hard palate medial vs. Hard palate lateral	27.73	Yes	*
Zygomatic bone vs. Hard palate lateral	21.49	No	ns

## Data Availability

All data can be requested from the authors (Email: christian.deininger@hotmail.com).

## Abbreviations

CMF	Craniomaxillofacial fracture
CT	Computed tomography
EFF	External face fixator
K-wire	Kirschner wire
LIC	Low-income country
HIC	High-income country
